# The relationship between dependence on artificial intelligence and creative thinking dispositions among graduate students in sport sciences

**DOI:** 10.3389/fpsyg.2026.1873886

**Published:** 2026-07-02

**Authors:** Didem Yavuz, Mehmet Fatih Karahüseyinoglu, Ayberk Kirdemir

**Affiliations:** Department of Physical Education and Sports, Faculty of Sport Sciences, Firat University, Elazig, Türkiye

**Keywords:** artificial intelligence dependence, creative thinking, education, human-AI interaction, sports sciences

## Abstract

**Introduction:**

The increasing prevalence of artificial intelligence (AI) technologies in higher education environments has significantly transformed the learning and research processes of graduate students in particular. While AI-supported tools accelerate access to information and facilitate academic processes, various discussions have arisen regarding the potential effects of excessive reliance on these technologies on higher-order cognitive skills, especially creative thinking. In this respect, the aim of this research is to examine the relationship between AI dependency and creative thinking dispositions among graduate students studying in faculties of sports sciences.

**Methods:**

In this research, a correlational survey model from quantitative research approaches was used. The population of the study consisted of graduate students studying in the faculties of sports sciences of eight universities located in the Eastern Anatolia Region of Türkiye. Within the scope of the study, data were collected from 286 graduate students using a convenient sampling method. The data were collected using the Dependence to Artificial Intelligence Scale and the Marmara Creative Thinking Dispositions Scale and analyzed using descriptive statistics, independent samples t-test, and Pearson correlation analysis.

**Results:**

The research findings revealed that participants’ dependence on artificial intelligence varied significantly depending on the AI tools they used most frequently. It was found that this difference favored participants who used personal assistants. Additionally, a statistically significant negative correlation was found between AI dependence and dispositions toward creative thinking.

**Discussion:**

These results highlight the importance of the balanced and mindful use of AI tools in graduate education programs for the sustainability of creative thinking skills.

## Introduction

The rapid developments in digital technologies have fundamentally transformed individuals’ daily lives, learning processes, and ways of accessing information. Artificial intelligence (AI) technologies, which is one of the most striking components of this change, have begun to be widely used in many areas of life, especially with the emergence of generative AI models (Eğin et al., 2025). These technologies serve users for various purposes such as acquiring information, solving problems, providing entertainment, and even emotional support, taking human-technology interaction to a new dimension (Sethi and Jain, 2024; Ruggiano et al., 2021).

In recent years, the development of large language model-based tools, especially ChatGPT, has further expanded the areas of use of AI technologies and led to their rapid spread in educational environments. Thanks to functions such as providing quick access to information, generating text, making explanations, preparing assignments, and planning content, AI technologies have become tools that students intensively use in their learning processes (Hu, 2023; Cotton et al., 2023). While these developments offer significant opportunities that can support academic performance (Alneyadi and Wardat, 2023; Chen et al., 2023), they have also led to the emergence of various risks such as addiction, social withdrawal, and loneliness as a result of the overuse of technologies (Xie et al., 2023; Laestadius et al. (2022).

AI technologies have the potential to support individuals’ learning processes, facilitate access to information, and enhance academic productivity (Marzuki et al., 2023; Başaran and Yeşilbaş Özenç, 2024; Karahüseyinoğlu et al., 2025). However, in recent years, researchers have also begun to discuss the potential effects of the intensive use of these technologies on individuals’ learning habits, decision-making processes, and their dependence on technology. Interaction between students and faculty in learning environments is being influenced by artificial intelligence, a situation that creates both opportunities and various concerns for higher education (Seo et al., 2021).

It is possible to state that one of these concerns is dependence on artificial intelligence. In this context, dependence on artificial intelligence can be conceptualized as an individual’s tendency to place a high degree of trust in and rely on AI systems during decision-making, task execution, or information verification processes. Advanced technologies such as AI can streamline processes and increase efficiency; however, they can also trigger feelings of insecurity and anxiety regarding the fear of being left behind if one cannot keep pace with these technologies (Morales-García et al., 2024). The concept of “AI dependence” used in this study does not refer to clinical dependence but rather to individuals’ perceived dependence on and trust in AI systems. Therefore, when evaluating the research findings, the concept must be interpreted not within the framework of clinical dependence but within the context of self-reported statements regarding AI usage.

The increasing role of artificial intelligence technologies in individuals’ learning processes has sparked new discussions regarding the use of higher-order cognitive skills. In particular, the potential effects of intensive AI technology use on processes such as independent thinking, problem-solving, and the generation of original ideas have drawn researchers’ attention in recent years. Consequently, the need to adopt a balanced approach to AI use and to support students’ psychosocial development is emphasized (Baron and Dajay, 2024).

Creative thinking refers to an individual’s capacity to develop original ideas, evaluate problems from different perspectives, and generate alternative solutions (Yeşilyurt, 2020; American Psychological Association, 2022). The creative thinking dispositions that form the foundation of this process consist of cognitive and behavioral characteristics, such as curiosity, perseverance, imagination, collaboration, and discipline, that guide the individual toward creative performance (Lucas et al., 2012).

At the graduate level, creative thinking skills are of critical importance for enhancing scientific productivity and ensuring the generation of original knowledge. Graduate students are expected not only to utilize existing knowledge but also to develop new ideas, approach research problems from different perspectives, and produce original work that contributes to the scientific field. The widespread adoption of artificial intelligence tools in academic research in recent years has heightened interest in the potential effects of these technologies on creative thinking processes. This is also significant for the field of sports science. In sports science, performance analysis, training planning, performance enhancement, post-injury recovery, tactical development, and research processes often involve complex problems that do not have a single correct answer and require the evaluation of different solution paths. Therefore, it is possible to state that a tendency toward creative thinking is an important component of both academic and applied processes in the field of sports science.

The aim of this study is to examine the relationship between the dependence on artificial intelligence and creative thinking dispositions among graduate students studying in the field of sports science. Given that the use of artificial intelligence is becoming increasingly widespread in higher education today, elucidating this relationship may contribute to understanding the potential effects of artificial intelligence technologies on students’ cognitive processes.

## Methods

### Research model

In this study, a correlational survey model was used within the scope of quantitative research approaches to determine the AI addiction levels and creative thinking tendencies of graduate students studying in the Faculties of Sports Sciences. The correlational survey model is defined as a research design that aims to reveal the direction and level of the relationship between two or more variables (Karasar, 2018).

### The population and sample of the study

The population of the study consists of postgraduate students studying at Faculties of Sports Sciences in the provinces of the Eastern Anatolia Region, which are located at the TRB1 (Malatya, Elazığ, Bingöl, Tunceli) and TRB2 (Van, Muş, Bitlis, Hakkâri) levels according to the regional classification created under the coordination of the State Planning Organization (DPT) and the Turkish Statistical Institute (TÜİK) within the scope of the Nomenclature of Territorial Units for Statistics (NUTS). The study population comprised postgraduate students studying in Faculties of Sport Sciences. A total of 286 participants were reached and voluntarily participated in the study during the data collection period. Convenience sampling was used in sample selection. The main reason for choosing the convenience sampling method is that reaching postgraduate students in all provinces throughout Türkiye involves difficulties in terms of time, cost, and access. Participants were reached both face-to-face and through an online survey form prepared via Google Forms. However, the inclusion criteria for the study were defined as follows: being an active graduate student at a Faculty of Sports Sciences, being over 18 years of age, voluntarily agreeing to participate in the study, and completing the data collection instruments in full. Forms that were incomplete or incorrectly filled out were excluded from the study.

The sample size of the study was determined using *a priori* power analysis conducted prior to the data collection process using the licensed G*Power 3.1.9.7 program. The analysis was based on the following parameters for the independent samples *t*-test: a moderate effect size (Cohen’s *d* = 0.50), a significance level (*α* = 0.05), and statistical power (1 − *β* = 0.95). The analysis determined that a minimum of 210 participants would be sufficient. The fact that 286 participants were recruited for the study indicates that the study exceeded the planned sample size and possesses sufficient statistical power.

### Data collection tools

In the study, in addition to an 6-question personal information form prepared to measure the demographic information of the participants, the “Dependence to Artificial Intelligence Scale” consisting of 5 questions and the “Marmara Creative Thinking Dispositions Scale” consisting of 25 questions were used.

### Dependence to Artificial Intelligence Scale (DAIS)

For data collection, the Turkish adaptation of the Dependence to Artificial Intelligence Scale, which was developed by Morales-García et al. (2024) and adapted into Turkish by Savaş (2024), was used. The scale consists of a 5-point Likert scale ranging from “strongly agree” (5) to “strongly disagree” (1). It was found that the scale’s Cronbach’s alpha internal consistency coefficient was 0.82, and the corrected item-total correlation values of the scale items ranged from 0.583 to 0.642. A high item-total correlation indicates that the scale has high internal consistency (Büyüköztürk, 2023). The lowest possible score on the scale is 5, and the highest is 25. There are no reverse-scored items on the scale (Savaş, 2024). In this study, a reliability analysis was conducted for the entire scale, and the Cronbach’s Alpha internal consistency coefficient was calculated as 0.83.

### Marmara Creative Thinking Dispositions Scale (MCTDS)

Developed by Özgenel and Çetin (2017), the creative thinking dispositions scale consists of 25 items and 6 sub-dimensions using a five-point Likert scale. The sub-dimensions are: “Self-discipline: 1, 6, 7, 15, 23”, “Novelty Seeking: 2, 5, 8, 12, 17, 19, 22, 24”, “Courage: 9, 11, 14, 25”, “Curiosity: 3, 10, 21”, “Doubt: 4, 16”, “Resilience: 13, 18, 20”. The questions in the scale are answered from (1) never to (5) always. The internal consistency coefficient of the scale was calculated as 0.87. A high score obtained from each sub-dimension of the scale indicates that the individual possesses the characteristic evaluated by that sub-dimension. The scale also provides a total creative thinking dispositions score. When scoring the scale, the average of the sub-dimensions and the total score is calculated (Özgenel and Çetin, 2017). The subscales included in the scale, such as “self-discipline,” “curiosity,” “skepticism,” “courage,” and “flexibility,” are based on theoretical foundations in the literature that demonstrate creative thinking is not merely a mental process but also a disposition requiring motivational and behavioral control (Bentley, 2004; Torrance, 1972). In this study, a reliability analysis was conducted for the entire scale, and the Cronbach’s Alpha internal consistency coefficient was calculated as 0.91.

### Data collection

The study was conducted with the approval of the Fırat University, Ethics Committee for Social and Human Sciences Research, session number 2025/13, dated June 13, 2025. The data collection process was carried out both in-person and online (Google Form) methods at the Faculties of Sports Sciences in the Eastern Anatolia Region. A total of 286 postgraduate students studying in the Faculties of Sports Sciences were reached within the scope of the research. Prior to the study, the purpose of the research was explained to the participants, and their informed consent was obtained. Participation was entirely voluntary, and participants were free to withdraw from the study at any stage. Data were collected anonymously and used solely for scientific purposes. No financial or non-financial incentives were provided to the participants.

### Data analysis

In this study, the levels of AI dependency and creative thinking dispositions of postgraduate students studying in the Faculties of Sports Sciences were examined. Normality distributions were examined to determine whether the data obtained from the scales and sub-dimensions met the normality assumption. For the Dependence to Artificial Intelligence Scale, the normality values were found to be Skewness = −0.626 and Kurtosis = −0.175. The normality values for the Creative Thinking Dispositions Scale are Skewness = −0.670 and Kurtosis = −0.086. The normality values for the sub-dimensions are as follows: Self-Discipline (Skewness = −0.305; Kurtosis = −0.518), Novelty Seeking (Skewness = −0.745; Kurtosis = 0.071), Courage (Skewness = −0.474; Kurtosis = −0.387), Curiosity (Skewness = −0.826; Kurtosis = 0.239), Doubt (Skewness = −0.494; Kurtosis = −0.195) and Resilience (Skewness = −0.914; Kurtosis = 0.347). These results indicate that all data are within the range of −1.5 to +1.5 and that the normality assumption is met (Tabachnick and Fidell, 2013). Homogeneity of variances, another assumption of parametric tests, was assessed using Levene’s test. The results indicated that the assumption of homogeneity of variances was met for the Dependence to Artificial Intelligence Scale, the Marmara Creative Thinking Dispositions Scale, and the vast majority of their subscales (*p* > 0.05). It was determined that the assumption of homogeneity of variances was violated only in the Courage subscale (*p* = 0.005). Therefore, the results of Welch’s *t*-test, calculated for unequal variances, were considered in the relevant comparison. Based on the findings, it was concluded that the use of parametric analyses was appropriate.

The analysis of the obtained data was performed using the licensed SPSS 27 software package. The independent samples t-test was used for the analysis of binary variables, and Pearson correlation analysis was used to examine the relationship between the Dependence to Artificial Intelligence Scale and the creative thinking disposition scale. The significance level was accepted as *p* < 0.05.

## Results

In this section, the findings obtained from the analysis of the data collected in accordance with the research objectives are presented in the form of tables accompanied by relevant explanations.

The distribution of participants according to their demographic characteristics is presented in Table 1. An examination of the sample reveals that the vast majority of participants hold a master’s degree and most frequently prefer research and chat applications (such as ChatGPT) among artificial intelligence tools. Notably, the gender distribution shows a higher proportion of male participants compared to females, while the age distribution is concentrated in the 21–28 age range. Additionally, nearly half of the participants reported using AI applications for an average of 1 h or less per day, while the majority of the sample stated that they generally find these applications reliable.

**Table 1:** Results regarding demographic data.

Variable	Groups	*n*	%
Educational status	Master’s degree	232	81.5
	Doctoral degree	52	18.5
Gender	Female	110	38.5
	Male	176	61.5
Age	21–24 years	98	34.3
	25–28 years	110	38.5
	29–32 years	37	12.9
	33 years and older	41	14.3
Duration of use	1 h or less	131	45.8
	2-3 h	119	41.6
	4 h and more	36	12.6
Perceived reliability of AI	Yes	191	66.8
	No	95	33.2
AI tools	Research and chat applications (ChatGPT vb.)	246	86.0
	Personal assistants (Siri vb.)	40	14.0
Total		286	100.0

Descriptive statistics for the scales are presented in Table 2. The minimum, maximum, mean, and standard deviation values for participants’ scores on the Dependence to Artificial Intelligence Scale and the Marmara Creative Thinking Disposition Scale are reported.

**Table 2:** Descriptive statistics of the scales.

Variables	Min.	Max.	*X¯*	Sd
DAIS	5.00	24.00	11.68	4.75
MCTDS	48.00	125.00	98.05	17.85

DAIS, Dependence to Artificial Intelligence Scale; MCTDS, Marmara Creative Thinking Dispositions Scale.

A t-test for independent samples was conducted to determine whether participants’ scores differed based on the type of AI tool they used most frequently, and the results are presented in Table 3. The findings indicate that participants who use personal assistants have higher AI dependency scores compared to those who use research and chat applications. Conversely, participants who use research and chat applications were found to have higher total scores for creative thinking dispositions, as well as higher scores in the dimensions of self-discipline, skepticism, and flexibility. When examining effect sizes, a small to moderate effect was observed for AI dependency and total creative thinking dispositions, a moderate effect for self-discipline and flexibility, and a relatively high effect for the skepticism dimension. No significant differences were found between groups in the dimensions of novelty seeking, courage, and curiosity.

**Table 3:** Independent samples *t*-test results by type of AI tool used which AI tools do you use most frequently?

Tool		*N*	*X¯*	Sd	*t*	*p*	*d*
DAIS	Research applications	246	11.43	4.59	−2.230	0.027	0.380
	Personal assistant	40	13.22	5.45			
MCTDS	Research applications	246	99.18	17.69	2.685	0.008	0.458
	Personal assistant	40	91.10	17.49			
Self-discipline	Research applications	246	19.58	3.86	2.948	0.002	0.503
	Personal assistant	40	17.67	3.32			
Novelty-seeking	Research applications	246	32.02	6.07	1.851	0.071	0.316
	Personal assistant	40	30.10	6.12			
Courage	Research applications	246	15.29	3.09	1.360	0.180	0.277
	Personal assistant	40	14.40	3.97			
Curiosity	Research applications	246	12.01	2.54	0.660	0.515	0.112
	Personal assistant	40	11.72	2.57			
Doubt	Research applications	246	8.06	1.44	5.061	<0.001	0.863
	Personal assistant	40	6.80	1.55			
Resilience	Research applications	246	12.21	2.42	4.356	<0.001	0.743
	Personal assistant	40	10.40	2.58			

The results of the Pearson correlation analysis are presented in Table 4. The findings indicate a significant negative relationship between AI dependency and creative thinking dispositions. Additionally, significant negative relationships were found between AI dependency and all subscales. These relationships generally range from weak to moderate.

**Table 4:** Correlation analysis.

Variables	r/p	DAIS
MCTDS	*r*	−0.332
	*p*	<0.001
Self-discipline	*r*	−0.332
	*p*	<0.001
Novelty-seeking	*r*	−0.256
	*p*	<0.001
Courage	*r*	−0.315
	*p*	<0.001
Curiosity	*r*	−0.280
	*p*	<0.001
Doubt	*r*	−0.201
	*p*	0.001
Resilience	*r*	−0.416
	*p*	<0.001

## Discussion

This study examined the relationship between AI dependency and creative thinking dispositions among graduate students enrolled in sports science departments. The findings presented in Table 1 indicate that participants had varying levels of experience with AI use. Furthermore, an analysis of usage duration revealed that a higher proportion of participants reported using AI for 1 hour or less. This finding suggests that participants generally use AI tools for short periods.

Today, students in particular devote a significant portion of their time to AI technologies. It has been observed that many students allocate between 1 and 2 hours, or more than 2 hours, to AI and its applications in their daily activities (Durmuş, 2025). Results from studies in the literature have shown that AI dependency scores increase as usage duration increases (Aydın, 2026; Erdmann et al., 2025). However, an increase in usage intensity should not be evaluated solely as an indicator of dependency behavior.

According to the findings in Table 1, the fact that the majority of participants find AI applications reliable indicates the presence of a positive perception toward these technologies. The fact that the most frequently used tools are research and chat-based AI applications (such as ChatGPT) suggests that participants prefer these types of tools for accessing information and academic processes. In contrast, the lower preference for personal assistant-type tools indicates that their usage frequency is more limited compared to other tools. Indeed, studies in the literature demonstrate that tools like ChatGPT provide significant support in research, data analysis, language learning, and academic production processes (Bukar et al., 2024; Farhi et al., 2023; Ahmadi and Tekemen, 2024).

According to the findings in Table 2, the average distribution of participants’ scores on the Dependence to Artificial Intelligence Scale within the sample is relatively low, while their scores on the creative thinking dispositions scale are relatively high. However, since there are no normative or clinical cutoff scores for the scales used, these results should be interpreted as relative distributions within the sample rather than categorical levels.

The fact that the research group consists of graduate students enrolled in sports science faculties can be considered a characteristic to be taken into account when interpreting this finding. The fact that individuals receiving education in the field of sports frequently encounter processes such as problem-solving, decision-making, and developing alternative strategies is among the characteristics that may be related to creative thinking skills. Although it may come to mind that this situation could stem from sample characteristics, it should not be forgotten that an empirical comparison with different disciplines has not been made.

In the literature, it is observed that artificial intelligence-related dependencies vary depending on different sample groups. For example, in a study conducted with students in the Medical Documentation and Secretarial Program, this finding was below average (Hoşgör and Gündördü, 2025), whereas among university students, it was reported to be close to or slightly above average (Estrada-Araoz et al., 2025; Kılıç and Koca, 2025). It is assessed that these score differences in the literature may be related to various individual and psychological dynamics, such as general perceptions of technology, as well as students’ academic self-efficacy beliefs or fears of being without artificial intelligence (AIlessphobia) (Gezgin and Türk Kurtça, 2025; Özbay, 2026). Indeed, some researchers note that students with relatively low academic self-efficacy or those who believe they would feel inadequate in academic tasks without AI support may turn to these tools more intensively, and this situation may increase their engagement scores (Acosta-Enriquez et al., 2025; Sarıkahya et al., 2025; Semerci Şahin et al., 2025). In this context, fluctuations in engagement averages observed across different samples may be related to the psychological needs or academic anxieties that drive students’ use of AI.

According to the findings in Table 3, comparisons based on the most frequently used AI tools by participants revealed that AI engagement scores showed significant differences depending on the type of tool used. This difference favors participants using personal assistants; in other words, individuals in this group have higher AI engagement scores. This finding suggests that the usage characteristics of different AI tools may be related to dependency. However, given the significant differences in sample sizes between the compared groups and the distinct functional characteristics of the tool categories, this finding must be interpreted with caution. A review of existing studies reveals that research directly examining variations in AI dependency based on the type of tool used is limited. Nevertheless, the literature indicates that individuals can engage in intense interactions with certain AI systems, and that such interactions may be associated with attachment dispositions over time (UNICEF, 2021; Wiederhold, 2018; Xie et al., 2023). In particular, the increasing role of AI-based tools in learning, entertainment, and social life has been assessed as potentially fostering dependency behaviors (Montag and Elhai, 2025).

According to the findings in Table 4, a negative relationship was identified between AI dependency scores and dispositions toward creative thinking. A review of the literature reveals that a study by Zhang et al. (2024) noted that excessive AI use may be associated with skills such as creativity, critical thinking, and autonomy. Furthermore, it is suggested that an excessive orientation toward AI may be linked to cognitive skills and motivation (Ahmad et al., 2023). Additionally, a systematic review by Zhai et al. (2024) indicated that excessive reliance on AI chat systems may be associated with cognitive skills such as critical thinking, analytical reasoning, and information evaluation. However, due to the cross-sectional nature of the current study, it is not possible to draw conclusions regarding the direction of this relationship.

When evaluated in terms of sub-dimensions, the findings presented in Table 4 reveal moderate, statistically significant negative relationships between AI dependency and self-discipline, courage, and flexibility. These results indicate an inverse relationship between AI dependency scores and self-discipline, courage, and flexibility. In particular, these negative relationships between AI dependency and traits related to cognitive and behavioral control—such as flexibility and self-discipline—suggest that there may be an inverse relationship between individuals’ self-regulation and adaptation skills and their use of AI. In contrast, weak but statistically significant negative relationships were found between AI dependency and the sub-dimensions of novelty seeking, curiosity, and skepticism. However, these dimensions are considered components of a creative thinking tendency within the theoretical framework of the scale used.

The fact that generative AI technologies, unlike traditional tools, not only support cognitive processes but also have the potential to replace these processes in some cases has sparked significant debates regarding the nature of creativity (Aru, 2025). It has been argued that the intensive use of AI tools in creative tasks may weaken individuals’ production processes based on their own internal knowledge and experience. This situation could carry the risk of reduced individual originality and more homogeneous creative outputs (Runco, 2023; Doshi and Hauser, 2024; Riedl and Bogert, 2024). Indeed, the widespread use of these same AI tools by many people can lead to a decrease in the diversity of creative outputs and an increasing similarity of ideas (Anderson et al., 2024; Moon et al., 2025). Additionally, the misuse or overuse of AI technologies may pose risks such as a decline in critical thinking and independent learning skills (Liu et al., 2023; Ray, 2023).

The concept of “dependency” addressed in the current study refers not to clinical addiction but rather to individuals’ intense orientation toward and tendency to use AI technologies. Therefore, it seems more appropriate to evaluate the findings within the framework of technology-oriented dependency and dispositions toward excessive use rather than behavioral addiction. Overall, the conveniences and efficiency advantages that AI technologies offer in educational processes cannot be overlooked. When the negative relationship identified in this study is evaluated alongside findings in the literature, it can be argued that a more detailed examination of how AI usage relates to creative thinking processes is necessary. Supporting the conscious and purpose-driven use of artificial intelligence in educational settings could contribute to the healthier management of its potential effects on students’ learning processes and creative thinking dispositions. Therefore, it is recommended that awareness programs regarding the use of artificial intelligence in educational settings be developed and that pedagogical approaches supporting students’ productive and responsible use of these technologies be adopted.

### Limitations

While this research offers significant contributions to understanding the relationships between AI addiction and creative thinking, it also has some limitations. First, the cross-sectional design of the research limits the causal interpretation of inferences regarding the direction of relationships between variables. Although the obtained relational patterns reveal possible interactions between variables, longitudinal designs are needed to explain how and over what time period AI addiction tendencies affect creative thinking processes.

Second, the fact that the data were collected using self-report-based measurement tools carries the risk of bias that may arise from how participants perceive their own behavior and cognitive tendencies. Particularly in areas requiring subjective evaluation, such as technology use and creativity, social desirability bias and errors in subjective assessment are important factors that must be considered when interpreting the results.

The limitation of the research group to individuals at a specific academic level may limit the generalizability of the findings to different age groups, disciplines, or professional fields. The fact that creative thinking processes can vary across disciplines and that AI usage habits are influenced by contextual factors necessitates conducting similar studies in different sample groups.

Furthermore, creative thinking tendencies were measured solely through self-report instruments. As a result, the findings should be interpreted as reflecting participants’ subjective evaluations rather than objective indicators of creative performance. Future studies may strengthen this line of research by incorporating performance-based and behavioral measures of creativity.

Finally, the present study relied on bivariate statistical analyses and did not control for potential confounding variables simultaneously. Future studies utilizing multivariate analytical approaches may provide a more precise understanding of the relationships between AI-related dependency and creative thinking tendencies.

### Implications

The results obtained show that policies and practices regarding the use of AI, especially in education and academic production processes, need to be reevaluated. It is crucial to develop structured learning strategies that encourage the conscious and purposeful use of AI tools in learning environments. In this context, it is necessary to develop not only students’ skills in using AI tools but also their competence in using these tools creatively. Furthermore, incorporating content that integrates AI literacy, critical thinking, and creative problem-solving skills into curricula can contribute to individuals being able to benefit from technological tools without becoming dependent on them. For institutions, developing guiding principles for the use of AI-supported systems and designing learning environments that support creative thinking can play an important role in reducing the potential risks of technology on cognitive development.

### Future research directions

The results of this research raise several research areas for a deeper examination of the relationships between AI addiction and creative thinking. First, it is important for future studies to examine how AI usage habits affect creative thinking processes over time using longitudinal research designs. Such studies will enable stronger inferences about the direction and continuity of relationships between variables.

Second, the use of experimental research designs can offer the opportunity to directly test the effects of AI usage intensity and usage patterns on creative thinking. In particular, experimental studies examining the contribution of human-AI collaboration to creative processes in different task types can more clearly reveal under what conditions technology supports or restricts creative thinking.

Finally, the use of neuroscientific and multi-method research approaches can contribute to a more comprehensive understanding of the differences between human creativity and AI- assisted production processes. Studies that combine brain imaging techniques, cognitive performance measurements, and behavioral data analysis will play a significant role in explaining the long-term effects of AI-assisted thinking processes on human creativity.

## Conclusion

This study shows that addiction tendencies toward the use of AI may be related to creative thinking. The findings reveal that how AI is incorporated into individuals’ thinking processes can affect the quality of creative thinking. In particular, it is believed that an excessive reliance on AI may reduce individuals’ active participation in thinking and limit creative skills such as generating new ideas, developing different solutions, and flexible thinking. This situation shows that AI is not only a helpful tool but also a technology that can influence thinking processes.

The rapid spread of AI technologies necessitates a reassessment of how creativity develops. AI tools offer significant opportunities for supporting creative processes and streamlining tasks. However, excessive and unconscious use of these tools can weaken individuals’ ability to generate their own original ideas and reduce the diversity of ideas. Therefore, in addition to the conveniences provided by AI, its long-term effects must also be considered.

Research results show that the effect of AI on creative thinking is not one-sided. It is understood that this effect can vary depending on how frequently AI is used, for what purpose it is used, and how actively the individual participates in the process. Therefore, rather than completely restricting the use of AI, a conscious and balanced approach to its use is considered a more appropriate strategy. Especially in education and academic processes, the use of AI as a supportive tool is important for individuals to maintain their creative production while preserving their own thinking skills.

In conclusion, this study highlights the relationship between AI addiction and creative thinking. As AI technologies become increasingly widespread today, how and within what boundaries these tools are used has become a significant issue. Achieving a balanced use between AI and human thinking abilities is of great importance for both individual development and the generation of original ideas.

## Data Availability

The raw data supporting the conclusions of this article will be made available by the authors, without undue reservation.
