# Correction: The relationship between dependence on artificial intelligence and creative thinking dispositions among graduate students in sport sciences

**DOI:** 10.3389/fpsyg.2026.1932993

**Published:** 2026-07-30

**Authors:** Didem Yavuz, Mehmet Fatih Karahüseyinoglu, Ayberk Kirdemir

**Affiliations:** Department of Physical Education and Sports, Faculty of Sport Sciences, Firat University, Elazig, Türkiye

**Keywords:** artificial intelligence dependence, creative thinking, education, human-AI interaction, sports sciences

An incorrect Funding statement was provided. The correct **Funding** statement reads:

The author(s) declared that financial support was received for this work and/or its publication. This research was supported by the Firat University Scientific Research Projects Coordination Unit under Project No. BSY.26.05.

The original version of this article has been updated.

